# Time-specific ultrasonic treatment of litchi thaumatin-like protein inhibits inflammatory response in RAW264.7 macrophages via NF-κB and MAPK transduction pathways

**DOI:** 10.1016/j.ultsonch.2023.106355

**Published:** 2023-03-04

**Authors:** Shiai Zeng, Kai Wang, Geyi Wu, Xuwei Liu, Zhuoyan Hu, Weichao Li, Lei Zhao

**Affiliations:** a Guangdong Provincial Key Laboratory of Food Quality and Safety, South China Agricultural University, Guangzhou 510642, PR China; b Intensive Care Unit, Sun Yat-sen Memorical Hospital, Sun Yat-sen University, Guangzhou 510120, PR China

**Keywords:** Ultrasound treatment, Litchi thaumatin-like protein, Molecular structure, Inflammation activity

## Abstract

The pro-inflammation activity of litchi thaumatin-like protein (LcTLP) led to be responsible for the occurrence of adverse reactions after excessive consumption of litchi. This study aimed to characterize the changes in the structure and inflammatory activity of LcTLP induced by ultrasound treatment. Significant molecular structure of LcTLP changes occured at 15 min ultrasound treatment, and then tended to recover with subsequent treatment. Secondary structure (*α-*helices decreased from 17.3% to 6.3%), tertiary structure (the maximum endogenous fluorescence intensity decreased), and microstructure (mean hydrodynamic diameter reduced from 4 μm to 50 nm) of the LcTLP treated for 15 min (LT15) were significantly affected, which led to the inflammatory epitope of LcTLP (domain II and V-cleft) unfolded. *In vitro*, LT15 had a significant anti-inflammatory response, which inhibited NO production and had the best effect at 50 ng/mL in RAW264.7 macrophages (73.24%). Moreover, proinflammatory cytokines such as tumor necrosis factor-*α* (TNF-*α*) and interleukin-6 (IL-6) secretion and mRNA expression levels were also significantly lower compared with untreated LcTLP (p < 0.05). Western blot further confirmed that the expressions of IκB-*α*, p65, p38, ERK and JNK reduced markedly (p < 0.05), which indicated LT15 inhibited the inflammatory response through NF-κB and MAPK transduction pathways. Overall, it can be hypothesized that LT15 exposed to low frequency ultrasonic fields have a direct effect on the protein surface structure and thus on the entry of LT15 into cells, making 15-minute ultrasound treatment potentially useful in reducing the pro-inflammatory properties of litchi or related liquid products.

## Highlights

• LcTLP treated by ultrasound for 15 min (LT15) altered multi-level structure.
• LcTLP structure tended to recover with ultrasound treatment reached 45-min.
• The inflammatory epitopes of LcTLP were disrupted at 15 min.
• Proinflammatory cytokines secretion and mRNA expression levels reduced by LT15.
• LT15 inhibited the inflammatory response through NF-κB and MAPK pathways *in vitro*.

## Graphical abstract

**Figure:**
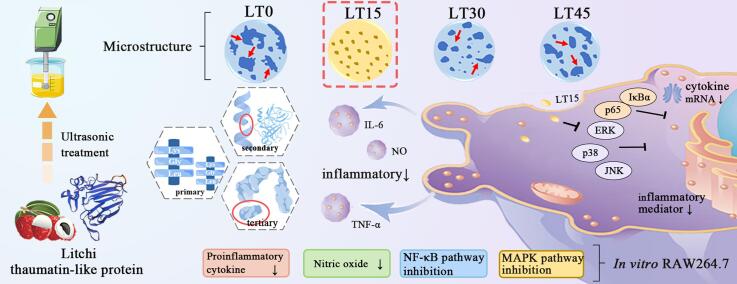

## 1 Introduction

Litchi (*Litchi chinensis* Sonn*.*) is a sweet and juicy subtropical fruit with various health-promoting effects (antioxidant, immunomodulation, and antihyperlipidemia) that is favored by many people [1], [2]. However, since ancient times, consuming too much litchi has frequently resulted in adverse reactions, such as dyspnea, itching, and rashes [3]. The causes of the adverse reactions to litchi have been investigated, and early scholars focused on allergic reactions, suggesting that the presence of proteins such as profilin and triose-phosphate isomerase are responsible for the allergy [4]. Subsequently, Wang, Hu, Yan, Ma, and Deng [5] revealed that soluble proteins in litchi are the main cause of adverse food reactions based on inflammatory effects. In 2020, a proinflammatory protein in litchi was discovered by our team and identified as a litchi thaumatin-like protein (LcTLP), and this protein significantly increased the levels of proinflammatory cytokines, such as tumor necrosis factor-*α* (TNF-*α*), interleukin-1*β* (IL-1*β*) and interleukin-6 (IL-6), in RAW264.7 macrophages [6]. Further studies conclusively showed that LcTLP was not fully digested and absorbed when it entered the gastrointestinal tract and caused changes in the intestinal flora and metabolites in the colon, ultimately causing liver inflammation via the gut-liver axis [7]. Consequently, the adverse effects of excessive litchi consumption are due to an inflammatory response caused by LcTLP-related food intolerance.

At present, the major way to avoid inflammation caused by litchi is to limit consumption, which seriously limits the development of litchi and related products [8]. Considering the need to retain or enhance the flavor, nutritional and health-promoting effects of litchi, safe and reliable processing strategies are being urgently sought and explored [9]. It is essential to consider whether processing might lessen the proinflammatory effects of litchi. In particular, using dry thermal processing to imitate the Maillard reaction in LcTLP, which modified its structure to decrease inflammatory activity, demonstrated the reason for the reduced inflammatory activity of dried litchi products [10]. This finding suggested that the adverse effects of overconsumption of lichi can be ameliorated by processing.

Low-frequency, high-intensity ultrasound (≤0.1 MHz, 10–1000 W/cm^−1^) is considered a promising nonthermal technology and is widely used in the food industry due to its ability to maintain the original freshness and nutrient content in food products [11]. Numerous studies have been conducted on the use of ultrasound in juices, meats, cereals and fermented products, where it not only inactivates enzymes and different food-borne pathogens but also enhances the color of the products and increases their stability during storage [12]. In addition, it has been reported that proteins can be modified by ultrasound treatment, and the cavitation effect of ultrasound affects biological functions by changing the spatial conformation of proteins [13], [14]. With these two concepts in mind, this property of ultrasound has been used to improve the allergenic properties of foods, including fruits, seafoods and dairy products [15], [16], [17]. Under ultrasound conditions of 20 kHz and 400 W, a 16-minute treatment reduced the protein content of the allergen Act d2 in kiwifruit by 50%, and similarly, a 20-minute treatment disrupted or split the primary structure of the shrimp allergenic protein molecule, resulting in a 76% reduction in allergenicity [18], [19]. In summary, ultrasound causes disruption (exposure or concealment) of the epitopes of allergenic proteins, thereby reducing their binding to IgE or decreasing their oligomerization levels, which affected their ability to trigger mast cell degranulation and ultimately reduced allergies [20], [21].

Thus, it is conceivable that the cavitation effect generated by ultrasound may lead to changes in the structure and proinflammatory activity of LcTLP during the ultrasound process. Therefore, in this study, the molecular chain and spatial structure of LcTLP after ultrasound treatment for different times were examined. Furthermore, the best fractions obtained were evaluated *in vitro* for inflammation using RAW264.7 macrophages to elucidate whether ultrasound could reduce the proinflammatory activity of LcTLP and explain the mechanism of the reduction in proinflammatory activity. This study aimed to provide a new approach and theoretical basis for addressing the adverse effects caused by litchi consumption.

## 2 Materials and methods

### 2.1 Materials and chemicals

Litchis (*L*. *chinensis cv.* Jizuili) were purchased from Guangzhou Jiali Dried and Fresh Fruit Food Co., Ltd (Guangzhou, China). The enzyme-linked immunosorbent assay (ELISA) test kits for TNF-*α*, IL-6 and TGF-*β*1 were purchased from Neobioscience Technology Co. (Shenzhen, China). Primary antibodies and secondary antibodies were purchased from Cell Signaling Technology (Danvers, MA, USA).

### 2.2 Sample preparation and ultrasound processing

LcTLP was extracted and purified from the fresh litchi pulp referred to the previous study [6]. 30 mg LcTLP was dissolved in 10 mL distilled water; place the solution in a 50 mL beaker and place in an Ultrusonic Cell Disrupter System (tip diameter 6 mm) (VCX 800, Sonics, America) for ultrasound treatment and cooled down using an ice bath. The ultrasonic output was set at 500 W and the ultrasound time was set at 0 (LT0), 15 (LT15), 30 (LT30) and 45 min (LT45), with pulse durations of 5 s on and 1 s off. All treatments and analyses were performed in triplicates.

### 2.3 Structural characterizations of ultrasonic treated LcTLP (U-LcTLP)

#### 2.3.1 SDS-PAGE

SDS-PAGE was performed in reducing condition. Briefly, the proteins were stacked on 5% (w/v) acrylamide gel and resolved on 12% (w/v) acrylamide gel for electrophoresis (Bio-Rad, U.S.A.), first at 80 V until the samples had entered the resolving gel and then at 120 V.

#### 2.3.2 Carbonyl content determination

The carbonyl content in the samples was measured by the 2,4 dinitrophenylhydrazine (DNPH) method [22], with slight modifications. The sample (3 mL) was reacted with 1 mL of DNPH for 30 min at room temperature. For the blank group, the DNPH solution was replaced by an equivalent volume of 2 mol/L HCl. After incubation, the DNPH-reacted sample were recovered by centrifugation after 20% trichloroacetic acid precipitation, and washed 3 times with an ethanol/ethyl acetate (1:1, V/V) mixture to remove unreacted DNPH. Finally dissolved in 3 mL of 6 M guanidine hydrochloride. Absorbance of protein was read at 370 nm. A molar absorptivity of 22,000 M^−1^ cm^−1^ was used to calculate protein carbonyl content and expressed as nmol/mg protein.

$$\mathrm{carbonylcontent}\left(nmol/mg\right)=\frac{\Delta A\times 10^{6}V_{1}}{22000\times cV_{2}}$$

Where ΔA is the different absorbance value between sample group and blank group, V_1_ is the volume of guanidine hydrochloride, V_2_ is the volume of sample, c is the protein concentration.

#### 2.3.3 Circular dichroism (CD) spectra

The secondary structure of U-LcTLP was measured using a CD spectrometer (Chriascan, UK Applied Photophysics). The cuvette has an optical range of 0.1 cm, a scanning range of 190–250 nm with a speed of 100 nm/min. The data was analyzed by a CDNN [7].

#### 2.3.4 Fluorescence spectrum

200 μL protein sample (1 mg/mL) was used to evaluate endogenous fluorescence intensity due to different time ultrasound treatment by multimode microplate reader (SpectraMax i3x, Molecular Devices). The excitation wavelength at 295 nm and the emission silt was 5 nm. Scanning ranged from 320 to 500 nm.

#### 2.3.5 Surface hydrophobicity determination (H_0_)

Diluting the protein samples with 0.01 mol/L PBS (pH 7.4) to a final concentration of 0.125–1.000 mg/mL, respectively, and taking 0.1 mL of the diluted protein samples with 0.5 mL of 8 mmol/L ANS solution. The fluorescence intensity (FI) of the mixture was measured at 390 nm (excitation) and 470 nm (emission) using F4500 fluorescence spectrophotometer (Hitachi Co, Japan). The initial slope of the FI versus protein concentration (mg/mL) plot (calculated by linear regression analysis) was used as an index of H_0_.

#### 2.3.6 Microstructure observation

Evaluation of microstructural changes in U-LcTLP with different ultrasound treatment times. This was done by using a transmission electron microscope (TEM) (JEM-1200EX, Hitachi High-Technologies Corporation. Tokyo, Japan). Images of U-LcTLP were taken at 2050 × and 14000 × magnifications.

### 2.4 Measurement of anti-inflammatory activity of LT15

#### 2.4.1 Cell culture and cytotoxicity test

RAW264.7 cells (Cell Bank of Chinese Academy of Sciences) were cultured in DMEM supplemented with 10% heat-inactivated FBS, 1% penicillin–streptomycin solution and 1% glutamax with 5% CO_2_ at 37 °C. After incubating with LPS, LcTLP and LT15 (50, 100, 200 ng/mL) for 24 h, the supernatant was replaced with 200 μL/well of CCK-8 solution under shaded conditions at 37 °C using the CCK-8 assay. After 1 h incubation, absorbance at 450 nm was read using a microplate reader. The data is shown in Figure S1(Supplementary data).

#### 2.4.2 Expression of inflammatory cytokines in RAW 264.7 cell

LT15 and LcTLP at 50, 100 and 200 ng/mL as experiment group and 100 ng/mL LPS as positive control group. The supernatant of basolateral culture medium was collected for measurements of NO by NO assay kit and of TNF-*α*, IL-6 and TGF-*β*1 by ELISA kit. Three independent experiments were performed with three parallel wells in each experiment.

#### 2.4.3 Gene expression level analysis

RAW 264.7 cells were treated with 200 ng/mL of LcTLP and LT15 respectively and total RNA was extracted from the cells by adding TRIzol reagent, followed by assaying the concentration and purity of the extracted RNA, checking RNA integrity and constructing an RNA-seq transcriptome library. After quantification, the RNA sequences were determined using HiSeq xen and analyzed using the KEGG PATHWAY and GO databases.

#### 2.4.4 Real-time quantitative PCR (RT-qPCR) validation

RAW264.7 cells were incubated for 24 h and then treated with different concentrations of LPS, LcTLP, and LT15 for 12 h. The total RNAs from RAW264.7 cells were extracted by Trizol reagent, and specific primers were used to amplify the polymerase chain reaction [23]. RT-qPCR was conducted with an iQTM SYBR Green Supermix kit (BIO-RAD, U.S.A.). Nucleotide sequences of the specific primers were showed in Table S1 (Supplementary data).

#### 2.4.5 Western blot

RAW264.7 cells (1 × 10^6^ cells/well) were planted in 6-well plates. Cells were stimulated by LPS (100 ng/mL) and LcTLP and LT15 (50, 100, 200 ng/mL) for 2 h. The supernatant was discarded and 150 μL of cell lysate (with 1% PMSF, 1% protease inhibitor and 2% phosphatase inhibitor) was added to each well to extract total protein. The proteins were separated by SDS-PAGE and transferred to a nitrocellulose membrane by electroblotting. The membranes were blocked with 5% nonfat milk for 1 h. The membranes were probed with the primary antibodies at 4℃ overnight, washed with TBST, and incubated for 1 h at 37℃ with secondary antibodies. Finally, membranes were then detected using an ECL detection reagent, and the bands were collected by the gel imaging system and quantified by Western banding using Image J software.

### 2.5 Statistical analysis

All experiments have been repeated three times and all data results were processed using IBM SPSS Statistics Version 20.0 and are expressed as mean ± standard deviation. Significant differences were analyzed using Duncan's test. Different letters (a − f) in the bars mean significantly different (*p* < 0.05). Graphical abstract was created by Figdraw.

## 3 Results and discussion

### 3.1 Effect of different ultrasound treatment times on the primary structure of LcTLP

**Fig. 1:**
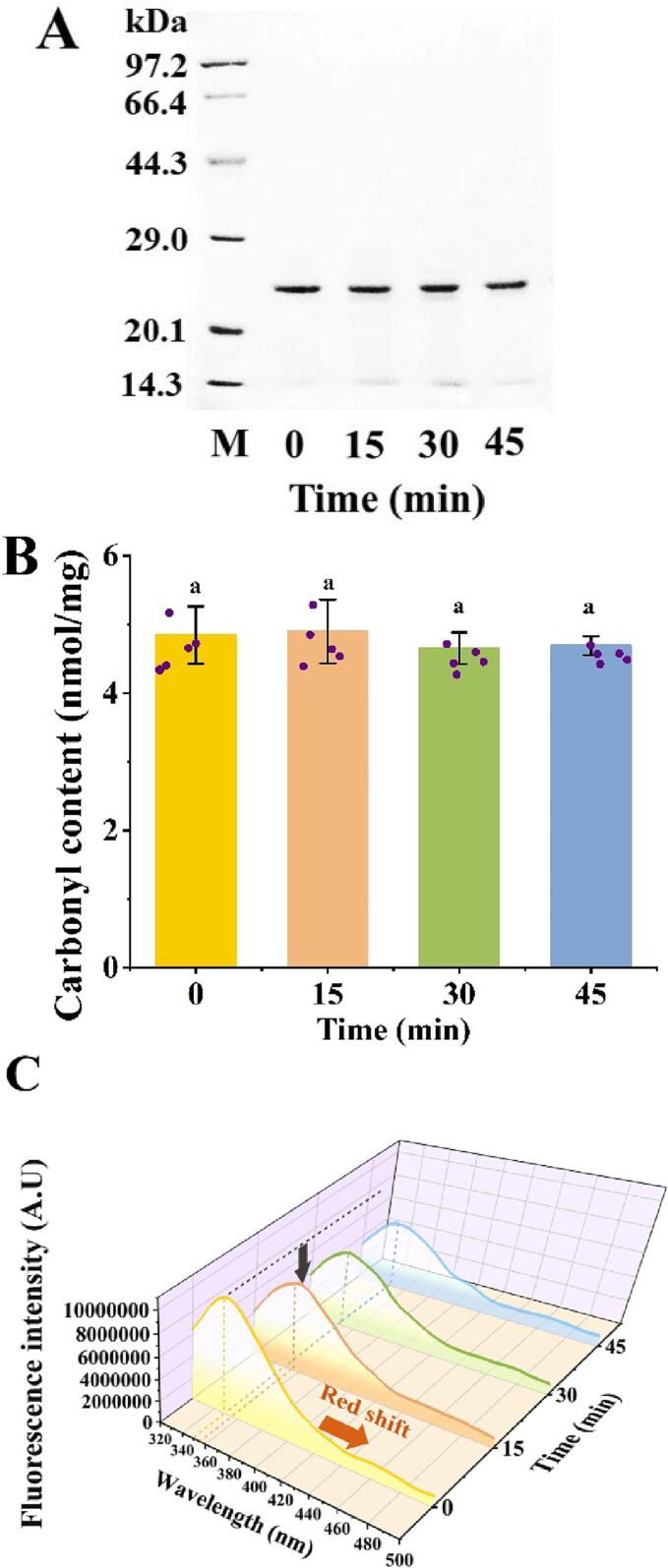

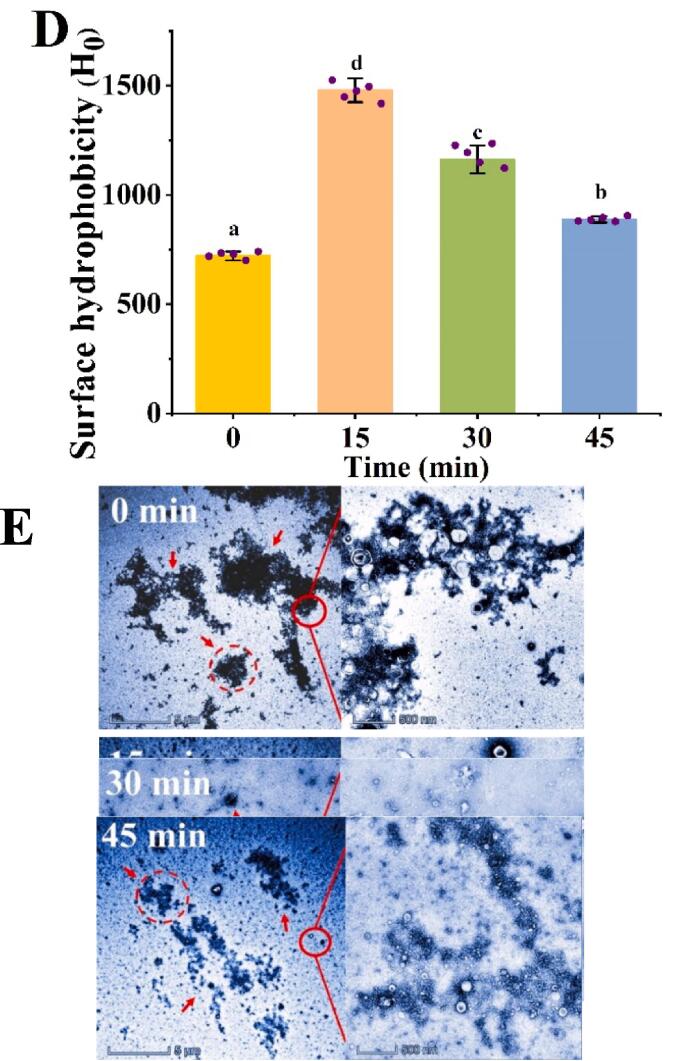
SDS-PAGE spectra (A), carbonyl content (B), endogenous fluorescence spectra (C), surface hydrophobicity (D) and transmission electron micrographs (E) of LcTLP treated with different ultrasound times.

The effects of different ultrasound treatment times on the primary structure of LcTLP were examined by SDS-PAGE and DNPH method (Fig. 1). All samples showed protein bands at the same position and intensity, indicating that no splitting of peptide bonds occurred during the sonication process (Fig. 1A). As shown in Fig. 1B, there was no significant difference in the carbonyl content between the treated and untreated samples, which demonstrated that ultrasound for different times did not break LcTLP peptide bonds or introduce exogenous carbonyl groups. The structural properties of LcTLP may help to protect the primary structure from the effects of ultrasound, as a previous study indicated that LcTLP was a protein aggregate with a molecular weight of 24 kDa that was stabilized by 8 disulfide bonds and was not susceptible to external stimuli such as enzymatic degradation, pH fluctuations and heat treatment [7]. These results were in agreement with the results for ultrasound treated walnut protein isolate and canola protein isolate [24], [25]. In conclusion, 500 W ultrasound treatment for 45 min did not cause changes in the primary structure of LcTLP. However, the effects on spatial structures need to be further explored.

### 3.2 Effect of different ultrasound treatment times on the secondary structure of LcTLP

CD spectroscopy is an analytical method for quantifying secondary structure changes in proteins [22]. The secondary structure changes caused by ultrasound treatment of LcTLP are shown in Table 1. The secondary structure of LT0 was found to have 17.3% *α*-helices, 36.6% antiparallel *β*-sheets, 6.2% *β*-sheets, 17.2% *β*-turns and 26.7% random coils before ultrasound treatment, which was similar to our previous study, and the only difference was an increase in the content of antiparallel *β* sheets (from 15.21% to 36.6%), which may be a change in sample state from solid to solution [6]. Some interesting changes in the secondary structure of LcTLP were also observed after ultrasound treatment, and there was a significant (*p* < 0.05) decrease in *α*-helices from 17.3% to 6.3%, a decrease in *β*-sheets from 6.2% to 3.8% and an obvious increase in anti-parallel *β*-sheets from 36.6% to 51.7% at 15 min. At 30 min, the secondary structure returned to its original state, and this restored structure did not appear to be altered by extending the treatment time. In particular, 15 min was the inflection point for structural changes throughout the ultrasound treatment.

**Table 1:** Relative content of the components of the secondary structure of LcTLP treated with different ultrasound times.

Ultrasound time	Secondary structure composition (%)				
(min)	*α*-helices	anti-parallel *β*-sheets	*β*-sheets	*β*-turns	random coils
0	17.3 ± 0.5^b^	36.6 ± 2.4^a^	6.2 ± 1.0^b^	17.2 ± 0.7^ab^	26.7 ± 2.3^a^
15	6.3 ± 0.7^a^	51.7 ± 2.3^b^	3.8 ± 0.5^a^	16.2 ± 0.8^a^	27.9 ± 2.3^a^
30	16.2 ± 1.4^b^	34.7 ± 1.2^a^	5.6 ± 0.6^b^	18.3 ± 0.4^b^	27.8 ± 1.5^a^
45	16.3 ± 0.8^b^	34.4 ± 1.1^a^	5.6 ± 0.5^b^	18.2 ± 0.8^b^	28.0 ± 1.4^a^

Equal letters in the same column indicate no statistically significant differences (*p* < 0.05).

After 15 min of treatment, *α*-helices decreased, and anti-parallel *β*-sheets increased, indicating that the protein was loosened, which was inseparable from the cavitation effect of ultrasound [26]. Local high temperatures, mechanical forces and free radicals generated by ultrasound treatment lead to partial disruption of the hydrogen bonds that stabilize the natural secondary structure of proteins, which can cause partial unfolding or breaking of the helix structure, resulting in denaturation and unfolding of the protein molecule and the dissociation of the original protein aggregates [25], [27]. Significantly, *α*-helices and *β*-sheets are the two main structures in proteins that are thought to be responsible for maintaining protein structure and influencing the biological activity of proteins [28]. In our previous study, LcTLP was found to be composed of three structural domains (I, II and III) and a V-cleft between structural domains I and II, where structural domain I formed the central flattened region of the protein and structural domains II and III were located on either side of the core structural domain [6]. Studies have shown that domain II and the V-cleft were the main inflammatory binding sites for thaumatin-like protein [29], [30]. It should be noted that 15 min of ultrasound led to changes in secondary structure, in particular the stretching of *α*-helices, disrupting helical domain II and further breaking the V-cleft. The changes in helical domain II and the V-cleft may further alter the inflammatory activity of LcTLP.

With prolonged ultrasound treatment time, the chance of collision with loose protein molecules and the attraction between protein molecules (e.g., hydrophobic interactions) both increased due to the unfolding of LT30. As the primary structure was not altered and the amino acid sequence was not disrupted, some denatured protein molecules may form new protein aggregates by interactions between peptide chains in the form of *β*-sheets, instead of forming as *α*-helices [31]. This finding also showed that the *β*-sheets content increased from 3.8% to 5.6% when the ultrasound time was extended to 30 min. In addition, the increase in *β*-sheets may lead to the formation of stronger hydrogen bonds between protein molecules, stabilization of the energy state, and a secondary structure does not change again with prolonged ultrasound time to 45 min [32].

### 3.3 Effect of different ultrasound treatment times on the tertiary structure of LcTLP

The chromophore is exposed to the solvent when the protein expands, leading to a decrease in fluorescence intensity, and tryptophan (Trp) is frequently utilized as an endogenous fluorescent probe for examining tertiary structures since it is more sensitive to changes in environmental polarity [33]. Intrinsic fluorescence spectra were used to analyze the phase changes in the tertiary structure of U-LcTLP at different ultrasound treatment times (Fig. 1C). LT0 had the highest maximum fluorescence intensity (FI_max_) (1 × 10^7^), which obviously decreased to 7 × 10^6^ after 15 min of ultrasound treatment. Further extension of the ultrasound time resulted in a moderate decrease in fluorescence intensity, with a FI_max_ of 6 × 10^6^ and 5 × 10^6^ for LT30 and LT45, respectively. In addition, the maximum absorption wavelength (λ_max_) of the intrinsic fluorescence spectrum of the samples underwent a slight redshift from 350 nm to 355 nm after 15 min of ultrasound treatment, but λ_max_ returned to its original position at 30 min and remained stable thereafter. The changes in intrinsic fluorescence were closely related to the structural changes in the protein during ultrasound treatment [34]. On the one hand, the cavitation and mechanical effects due to ultrasonic irradiation caused severe damage to the LcTLP molecule, leading to unfolding of the LT15 structure (a decrease in *α*-helices) and giving the tryptophan chromophore the opportunity to be more exposed to the hydrophilic medium, thus promoting collision bursts and rapidly reducing the quantum yield of fluorescence [35]. On the other hand, a redshift in λ_max_ was observed for LT15, indicating an increase in the polarity of the Trp residue microenvironment. The V-cleft of the thaumatin-like protein had been reported to be highly electronegative, and so the altered microenvironment polarity of Trp residues may be due to ultrasound-induced unfolding of the V-cleft of LcTLP, freeing the electronegative ions into the solution [36]. Unusually, the reduction in FI_max_ after 30 min was gentler than at 0–15 min, which may possibly due to the fact that after 15 min the overall energy of the chromophore was impaired. The exposure of non-polar side group residues in the unfolded protein triggers protein aggregation, resulting in an increase in steric hindrance. Similarly, LT30 shifted the λ_max_ from 355 nm to 350 nm, which indicated that Trp was buried and that the tertiary structure of LcTLP was rearranged, providing another demonstration of U-LcTLP reaggregation [37], [38]. Thus, different ultrasound treatment times altered the tertiary structure of LcTLP, showing a trend of stretching first and then clustering after 15 min with increasing ultrasound treatment times, which is consistent with the results for the secondary structure.

An extrinsic fluorescent probe (ANS) was then applied to determine the surface hydrophobicity (H_0_) of the protein. As expected, the changes in surface hydrophobicity were similar to the result of intrinsic fluorescence (Fig. 1D). From 0 to 15 min, H_0_ increased from 719.71 to 1479.10. This was possibly indicated by ultrasonically induced cavitation leading first to the cleavage of hydrophobic bonds and an increase in exposed surface area, which further led to protein denaturation and facilitated the release of hydrophobic amino acids from the core [39]. With increasing ultrasound treatment time, H_0_ eventually decreased to 887.45. The reduction in surface hydrophobicity is a sign of protein aggregation, which protects the hydrophobic regions of U-LcTLP [40]. By prolonging the ultrasound treatment time (>15 min), the internal molecules promoted noncovalent bonding to exposed regions, resulting in the formation of aggregates in the network and reduced hydrophobicity [40]. Interestingly, unlike secondary structure changes, 45 min of ultrasound treatment did not restore the tertiary structure changes to the untreated structure. It is possible that LT15 was subjected to ultrasonic cavitation, that temperature and ionic strength led to a break in noncovalent bonds, the broken bond reassociated with the side chains of amino acid residues in the new environmental regime, and the polypeptide chains enfolded to form new aggregates [32]. As a result, the three domains of LcTLP may be altered to varying degrees, and at 15 min, the binding site associated with inflammation may be further affected due to loosening of the structural domains and V-cleft stretching.

### 3.4 Effect of different ultrasound treatment times on the microstructure structure of LcTLP

To further visualize the effect of ultrasound treatment on the structure of LcTLP, transmission electron microscopy was performed (Fig. 1E). The microstructure of natural LcTLP was large and aggregated, with an average hydrodynamic diameter of the minimum aggregate of approximately 4 μm and irregularly contoured edges (red arrow and dashed line circle). After 15 min of ultrasound treatment, a clear dispersion of LT15 occurred, mainly in the form of small, uniformly distributed spheres with a diameter of 50 nm (red dashed line circle). As the ultrasound time continued, LT30 formed spherical aggregates with a diameter of 100 nm or more (red dashed line circle), and when the ultrasound treatment was complete, LT45 formed irregular aggregates with a 4 μm hydrodynamic diameter (red dashed line circle), although the degree of aggregation did not reach that of the untreated condition. These results further confirmed the changes in secondary and tertiary structures by prolonging the ultrasound treatment time. Ultrasound has considerable mechanical power to disrupt molecular interactions and swell proteins, as well as to reduce protein aggregation [41]. However, during prolonged ultrasound treatment, this process may be reversed, as characterized by enhanced protein aggregation. In fact, Chandrapala [40] demonstrated that recombinant whey protein increased the denaturation enthalpy due to protein aggregation after 5 min of ultrasonic treatment. In addition, Wang [20] found a significant correlation between casein particle size and allergies. Casein colloids with diameters<100 nm after the ultrasound process (25 kHz, 900 W for 60 min) showed low allergenicity due to the disruption of conformational epitopes, which significantly reduced degranulation induction and reduced IgE binding capacity. Therefore, whether changes in the structure of the small particle size LT15 after ultrasound treatment will correspondingly alter proinflammatory activity needs to be explored.

### 3.5 Effects of LT15 on the production of NO and inflammatory cytokines in RAW264.7 macrophages

**Fig. 2:**
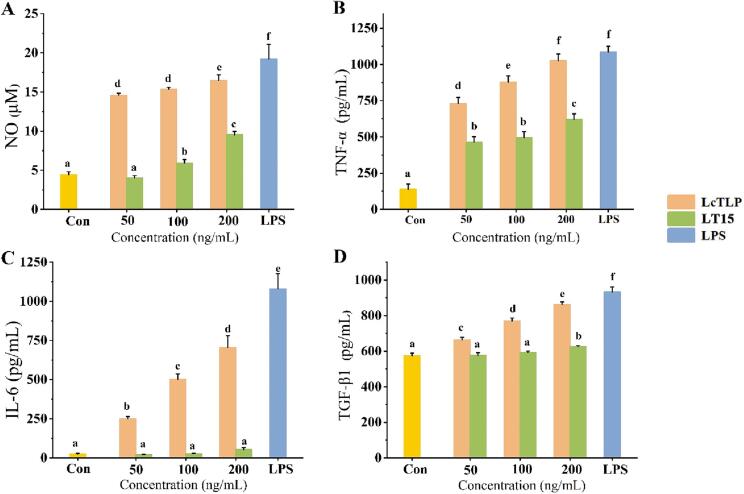
The inflammatory activity of LT15 and LcTLP. (A) NO content. (B) The secretion level of TNF-*α*. (C) The secretion level of IL-6. (D) The secretion level of TGF-*β*1.

Previous studies have shown that LcTLP caused inflammatory responses *in vivo* and *in vitro* [7]. To detect the effect of LT15 on the cellular inflammatory response after ultrasound, inflammatory activities were measured, and the results are shown in Fig. 2. Overall, the secretion of NO and the inflammatory cytokines TNF-*α*, IL-6 and TGF-*β*1 in LT15 were significantly (*p* < 0.05) lower than those in the LcTLP and LPS groups. As depicted in Fig. 2A, the NO secretion by LT15 stimulated in response to 50, 100 and 200 ng/ml was 4.01, 5.92 and 9.53 μM, resulted in reductions of 72.49%, 61.56% and 42.10%, respectively, relative to LcTLP, which was similar to that of LcTLP treated with the Maillard reaction [10]. NO acts as an important molecular signal in the inflammatory process and positively reflect the severity of inflammation [42]. Furthermore, TNF-*α* and IL-6 are important proinflammatory cytokines, and as expected, compared to LcTLP, LT15 caused 36.71%, 43.73% and 39.41% TNF-*α* inhibition and 91.96%, 94.64% and 92.51% IL-6 inhibition at 50, 100 and 200 ng/mL, respectively (Fig. 2B and 2C). The secretion of the anti-inflammatory cytokine TGF-*β*1 was significantly (*p* < 0.05) reduced by LT15, and there was no significant difference between LT15 at other concentrations and the control group, except at 200 ng/mL (Fig. 2D). TGF-*β*1, an anti-inflammatory cytokine, regulates the balance of the inflammatory response. These results suggested that LT15 attenuated the inflammatory response by reducing the production of NO, TNF-*α*, IL-6, and TGF-*β*1 by macrophages.

### 3.6 Effects of LT15 on the mRNA levels of inflammatory mediators in RAW264.7 macrophages

**Fig. 3:**
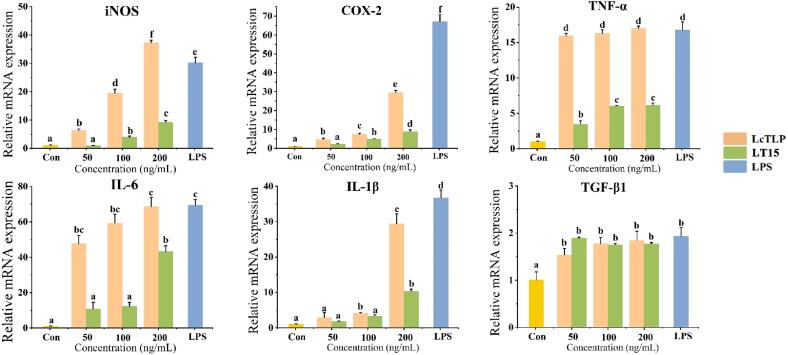
LT15 reduces the expression of genes involved in inflammation.

RT-qPCR was used to determine whether the LT15-induced reduction in inflammatory activity was due to the inhibition of inflammation-related genes. In this study, the transcription levels of all inflammatory mediators were suppressed by LT15 compared to LcTLP (Fig. 3). Inducible nitric oxide synthase (iNOS) transcription was positively correlated with NO production. Inhibition of iNOS expression resulted in a decrease in NO secretion, and the lowest expression of iNOS occurred in response to 50 ng/mL LT15 treatment, which was not significantly different from that in the control group and was consistent with the ELISA findings that NO secretion was also lowest. In addition, LT15 reduced the expression of cyclooxygenase-2 (COX-2) by 74.08%, 79.91% and 64.90% compared to the same concentration in the LcTLP group, respectively. Increased production of prostaglandin E_2_ (PGE_2_) by COX-2 is known to accelerate the inflammatory response by inducing the expression of various inflammatory cytokines, such as IL-1*β* and TNF-*α*, and NO production by iNOS also plays an important role in COX-2 expression [43]. The gene expression levels of IL-1*β*, TNF-*α* and IL-6 were also significantly lower in macrophages after LT15 treatment compared to LcTLP (*p* < 0.05). In addition to 50 ng/mL, LT15 had an inhibitory effect on the anti-inflammatory cytokine TGF-*β*1, but the effect was not significant. These results were consistent with the reduced secretion of proinflammatory and anti-inflammatory mediators in LT15-treated RAW264.7 cells. Consequently, it was determined that LT15 could reduce the proinflammatory activity *in vitro*.

The reduction in the proinflammatory effect of LT15 on RAW264.7 cells may be related to structural alterations. Studies have shown that domains II with *α*-helices and V-clefts in thaumatin-like proteins were epitope regions that were primarily responsible for inflammatory activity [30]. Combined with the secondary structure results, the *α*-helices content decreased from 17.3% to 6.3%, and domain Ⅱ was destroyed of LT15. Moreover, the tertiary structure was also disrupted. The H_0_ of LT15 increased from 719.71 to 1479.10, indicating that ultrasonic treatment increased hydrophobic bond breaking and exposed surface area, which further unfolded LT15 and disrupted the V-cleft between domains Ⅰ and Ⅱ, thus affecting the inflammatory binding site and reducing the proinflammatory activity of LT15.

### 3.7 Effect of LT15 treatment on the transcriptome of macrophages

**Fig. 4:**
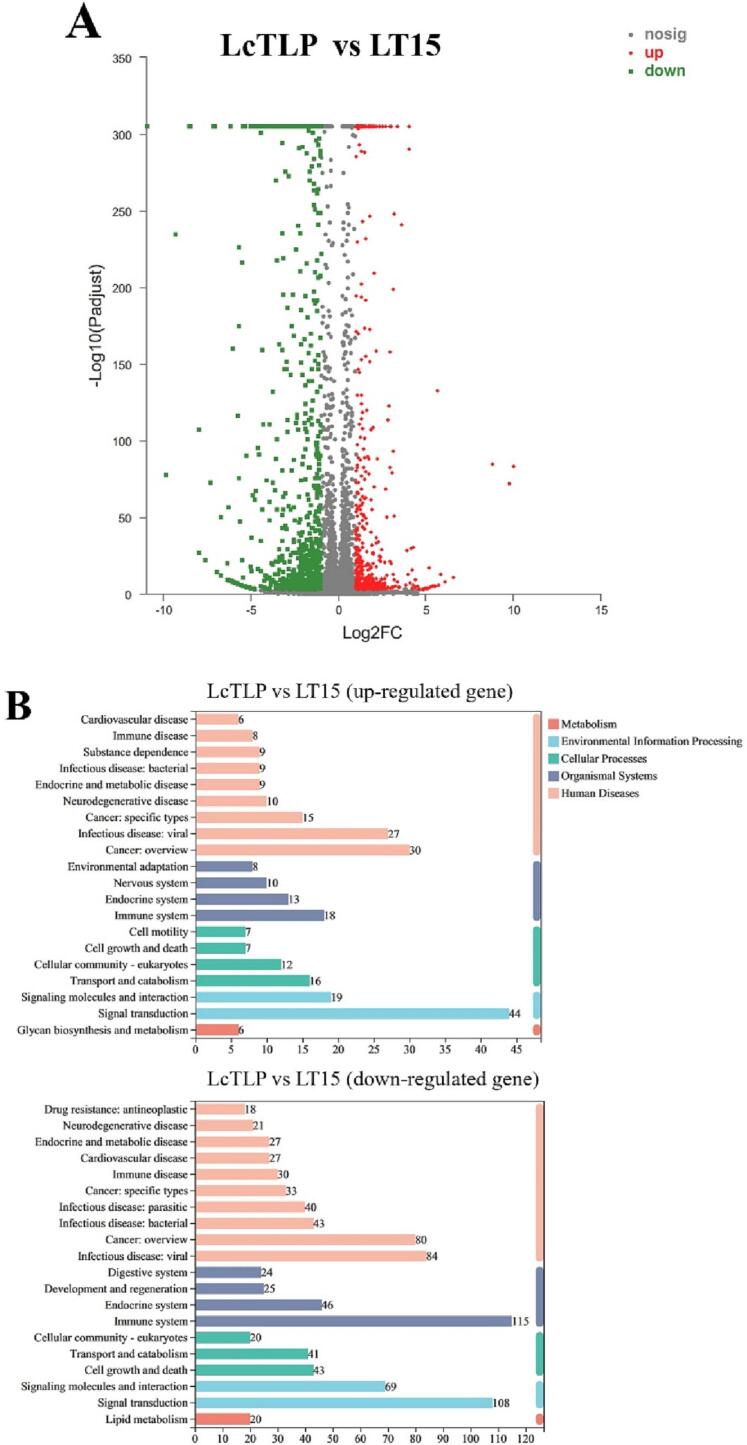

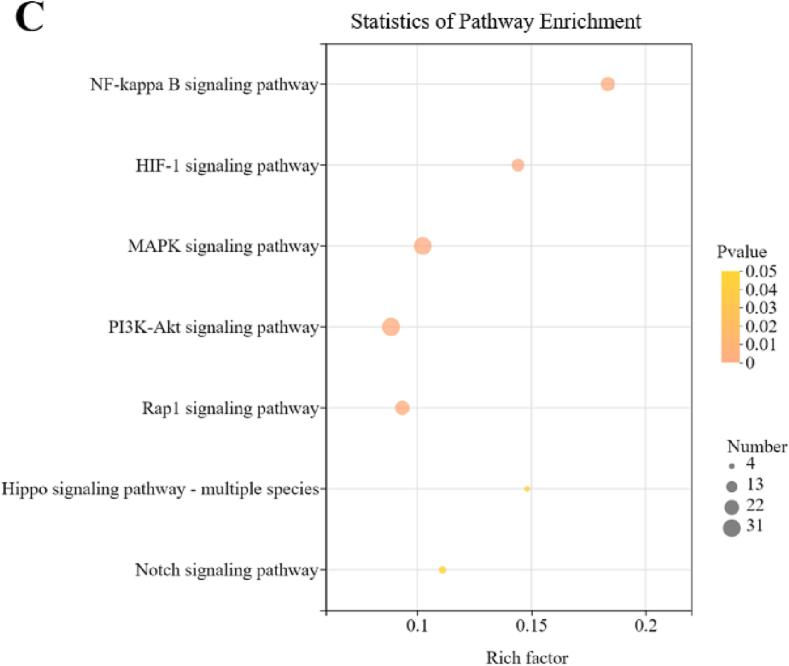
Gene Ontology enrichment analysis (A) Volcano plots of differentially expressed genes (DEGs). (B) KEGG function analysis of DEGs between LcTLP group and LT15 group. (C) KEGG pathway analysis comparing LT15 and LcTLP groups.

Total macrophage mRNA was extracted and reversed transcribed, and after PCR amplification, the obtained DNA was identified and annotated. Fig. 4A shows the volcano plots of differentially expressed genes (DEGs) of LT15 and LcTLP, and each point represents a specific gene. The red dots indicate significantly upregulated genes, the green dots indicate significantly downregulated genes, and the gray dots are nonsignificantly differentially expressed genes; dots in the upper left were more significantly expressed. Compared to the LcTLP group, LT15 stimulation of macrophages induced a total of 1368 differentially expressed genes, of which 484 genes were significantly upregulated and 884 genes were significantly downregulated, indicating that LT15 regulated the transcript levels in RAW264.7 cells after ultrasound and was similar to the gene expression of the blank group. Fig. 4B shows that the resulting differentially expressed mRNAs were subjected to KEGG annotation. The altered mRNAs were enriched in various pathways, including metabolism, environmental information processing, cellular processes, organismal systems and human diseases. LT15 significantly downregulated 115 genes of associated with the immune system and 108 genes associated with signal transduction in the LcTLP group, indicating that LT15 inhibited the proinflammatory effect of LcTLP on macrophages mainly through the regulation of these two functions. Specifically, signal transduction in environmental information processing is shown in Fig. 4C. LT15 significantly affected seven signaling pathways, including NF-κB, HIF-1, MAPK, PI3K-AKT, Rap1, Hippo and Notch (*p* < 0.05). Among them, NF-κB was the most enriched with a Rich factor of 0.18, suggesting that the attenuation of proinflammatory activity by LT15 may significantly regulate the NF-κB signaling pathway. In the bubble chart, a large bubble represented by the MAPK signaling pathway indicates a high number of candidate target genes. LT15 significantly altered 30 genes in the MAPK signaling pathway, which may include genes that attenuate the inflammatory response, as MAPK is a common pathway involved in the inflammatory cascade.

### 3.8 Effect of LT15 on the regulation of the NF-κB and MAPK signaling pathways

**Fig. 5:**
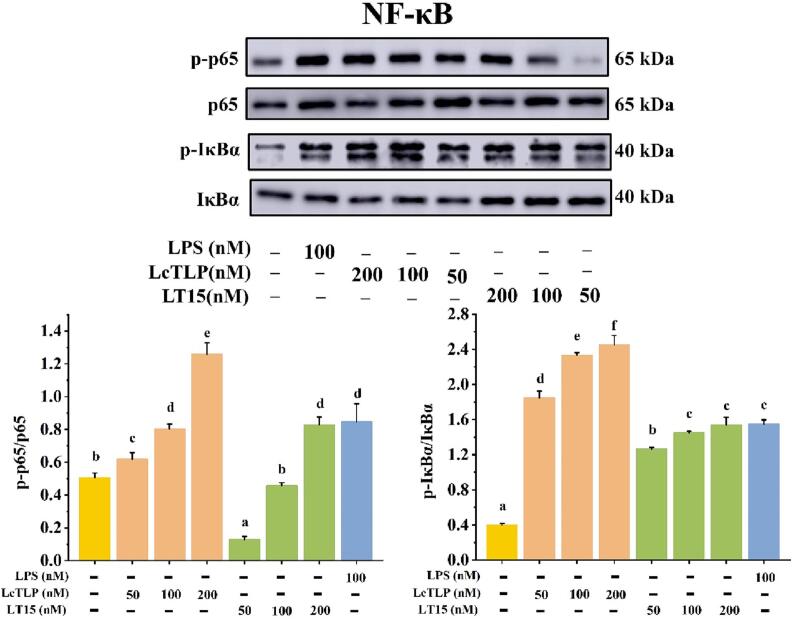
LT15 decreased the secretion levels of pro-inflammatory related protein in NF-κB pathway.

The phosphorylation levels of the LT15-stimulated downstream kinases p65, IκB*α*, p38, JNK, and ERK were examined by western blot analysis to further investigate whether the LT15-induced reduction in macrophage inflammation was mediated through the nuclear factor kappa-B (NF-κB) and mitogen-activated protein kinase (MAPK) pathways. The effects of LT15 and LcTLP on the expression of related activator proteins in the NF-κB pathway are shown in Fig. 5. The expression of p-p65/p65 and p-IκBα/IκBα was significantly enhanced by LcTLP compared to that in the control group, which was similar to a previous *in vivo* study [7]. However, LT15 could reverse this effect and reduce the inflammatory response. LT15 reduced the phosphorylation of p-p65/p65 by 79.17%, 43.21%, and 34.30% at 50, 100, and 200 nM, respectively, and p-IκBα/IκBα by 31.49%, 38.01% and 37.27%, demonstrating that LT15 decreased inflammation by inhibiting the activation of critical proteins in the NF-κB pathway: p65 and IκB*α*. In particular, the levels of p-p65/p65 were dramatically reduced by LT15 at 50 and 100 nM and were lower than those in the control group. This finding indicated that inflammation was decreased and that the protein expression level of p65 had returned to a normal level. In normal cells, the p50 and p65 subunits remain inactive in the cytoplasm by binding to IκB. In contrast, during inflammation, activation of IκB kinase (IKK) leads to the phosphorylation of IκB and the translocation of NF-κB, indicating activation of the NF-κB pathway [44]. Ultimately, NF-κB heterodimers are released into the cytoplasm, allowing them to translocate to the nucleus to bind to nucleotide sequences in the κB structural domain and regulate the transcription of various inflammatory cytokines and adhesion molecules [45]. Thus, LT15 inhibited the phosphorylation of IκB*α* and the nuclear translocation of p65, thereby inhibiting the expression of proinflammatory markers.

**Fig. 6:**
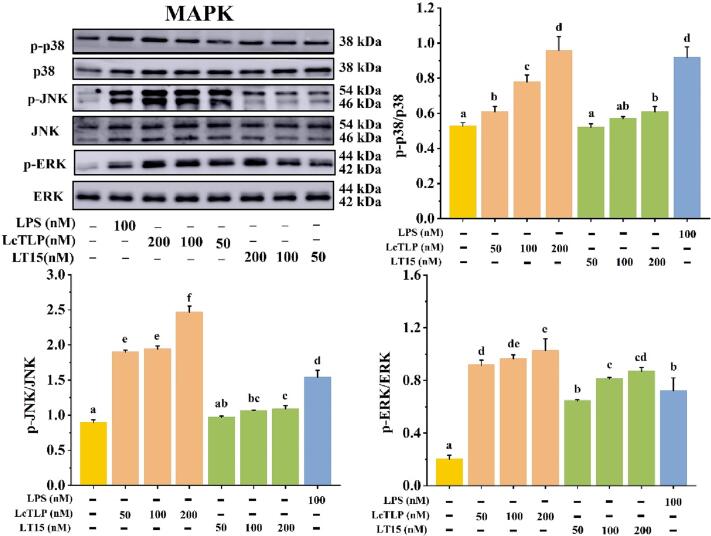
LT15 decreased the secretion levels of pro-inflammatory related protein in MAPK pathway.

Similar to NF-κB, MAPK is another fundamental signaling pathway involved in the regulation of proinflammatory cytokine expression during inflammation, and the production and release of multiple downstream transcription factors are mediated by three key members: p38, JNK and ERK [46]. MAPK is also involved in regulating NF-κB transcriptional activity, and p38 and JNK can induce IкB*α* degradation [47]. The effect of LT15 on activating proteins in the MAPK pathway is shown in Fig. 6. Compared to that in the LcTLP group, LT15 significantly (*p* < 0.05) inhibited the expression of the three upstream kinases of MAPK pathway activation. The degree of phosphorylation of p-p38/p38, p-JKN/JNK, and p-ERK/ERK by LT15 treatment decreased by 36.41%, 55.90% and 15.37%, respectively, compared with LcTLP at 200 nM. Although ERK, JNK and p38 are downstream factors of MAPK, the decrease in ERK was less significant than that of JNK or p38. This may be due to the TLR5-MyD88-IRAK1/4-TRAF6-TAK1-IKK*β* signaling pathway. IKK*β* first regulates IκB*α*, which is involved in ubiquitin-mediated proteolysis, while the downregulation of p105 was weak, the pathway regulating ERK was complex, and the inhibitory effect was not obvious (Fig. S2). The results showed that LT15 could affect the NF-κB pathway by inhibiting the phosphorylation of NF-κB p65 and IκB*α* in RAW264.7 cells and could affect the MAPK pathway by inhibiting the phosphorylation of JNK, ERK and p38, leading to changes in the secretion of inflammatory cytokines and enzymes.

## 4 Conclusion

In this study, the effects of ultrasound treatment on the primary structure, secondary structure, tertiary structure, microstructure and proinflammatory activity of LcTLP were assessed. 15 min was the most critical point associated with structural changes throughout the process, and the intense shear stress and pressure generated by ultrasound led to disruption of the secondary structure, tertiary structure and microstructure. Specifically, the inflammatory epitopes of LcTLP, helical domain II, and the V-cleft formed by the linkage between the domains were also inevitably disrupted during the unfolding of the structure at all levels. However, there was a tendency for the protein structure to recover with the extension of ultrasound treatment time. *In vitro* experiments showed that ultrasound treatment assisted in eliminating the pro-inflammatory activity of LcTLP. LT15 significantly inhibited the production of inflammatory cytokines and the activation of the NF-κB and MAPK signaling pathways. This finding suggests that ultrasound can be used as a green technology to address intolerance of litchi and its related products, thus providing a potential strategy to reduce the loss of value in the occurrence of adverse reactions to litchi consumption.

## CRediT authorship contribution statement

**Shiai Zeng:** Methodology, Investigation, Formal analysis, Writing – original draft. **Kai Wang:** Methodology, Writing – review & editing. **Geyi Wu:** Methodology, Investigation. **Xuwei Liu:** Formal analysis, Data curation. **Zhuoyan Hu:** Project administration, Funding acquisition. **Weichao Li:** Methodology, Writing – review & editing. **Lei Zhao:** Methodology, Writing – review & editing.

## Declaration of Competing Interest

The authors declare that they have no known competing financial interests or personal relationships that could have appeared to influence the work reported in this paper.
