# Supplementary material for: Time-specific ultrasonic treatment of litchi thaumatin-like protein inhibits inflammatory response in RAW264.7 macrophages via NF-κB and MAPK transduction pathways
Source: Ultrason Sonochem. 2023 Mar 4;95:106355. doi: 10.1016/j.ultsonch.2023.106355 (PMC10020100; doi:10.1016/j.ultsonch.2023.106355)
Supplement: Supplementary data 1 [file mmc1.docx]

**Supplementary Information**

**Time-specific ultrasonic treatment of litchi thaumatin-like protein inhibits inflammatory response in RAW264.7 macrophages via NF-κB and MAPK transduction pathways**

Shiai Zeng^1^, Kai Wang^1,2^, Geyi Wu^1^, Xuwei Liu^1^, Zhuoyan Hu^1,2^, Lei Zhao^1,2*^

^1^ College of Food Science, South China Agricultural University, Guangzhou 510642, P.R.China.

^2^ Guangdong Laboratory for Lingnan Modern Agricultural, Guangzhou 510642, P.R.China.

^*^Corresponding Author:

Tel: +86-20-85280295, Email: [scauzl@scau.edu.cn](mailto:scauzl@scau.edu.cn) (L. Zhao)

Table S1 Sequences of the primers used in quantitative RT-qPCR reactions

| Target gene | Forward (5*'*-3*'*) | Reverse (5*'*-3*'*) |
| --- | --- | --- |
| GAPDH | GTCATTGAGAGCAATGCCAG | GTGTTCCTACCCCCAATGTG |
| COX-2 | TGCACTATGGTTACAAAAGCTGG | TCAGGAAGCTCCTTATTTCCCTT |
| iNOS | AAGCAGCTGGCCAATGAG | CCCCATAGGAAAAGACTGCA |
| TNF-*α* | GGGAGCAAAGGTTCAGTGA | CCTGGCCTCTCTACCTTGTT |
| IL-6 | GCGATGGAACTTCGACTTTGT | GGGCTTCCTCTTGGAGAAGAT |
| IL-1*β* | GAGCCTGTGTTTCCTCCTTG | CAAGTGCAAGGCTATGACCA |
| TGF-*β*1 | CTTCAATACGTCAGACATTCGGG | GTAACGCCAGGAATTGTTGCTA |


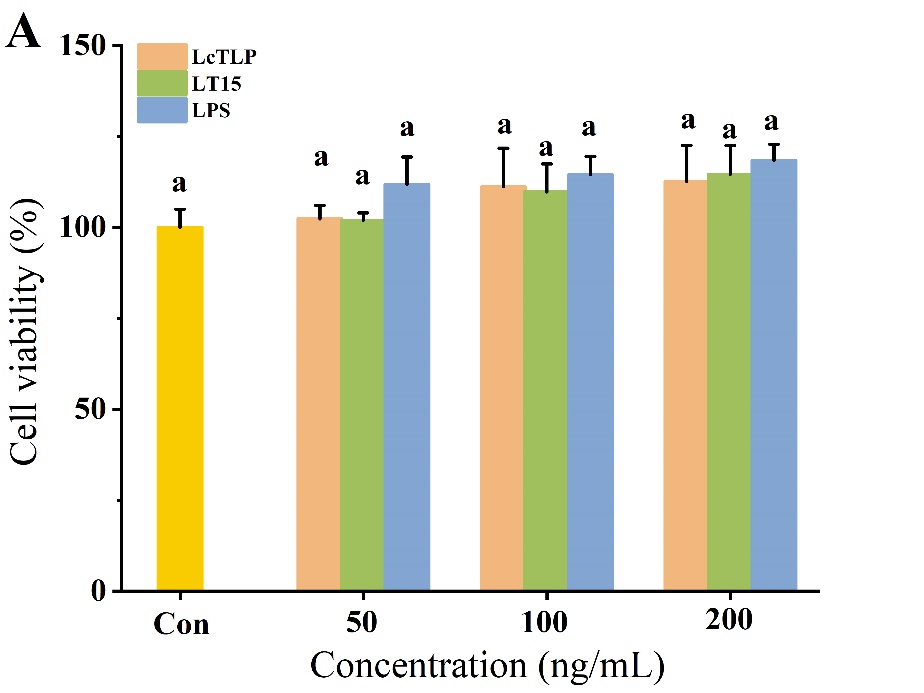


Fig. S1. Cell viability of LT15, LcTLP and LPS in different concentrations on cells.


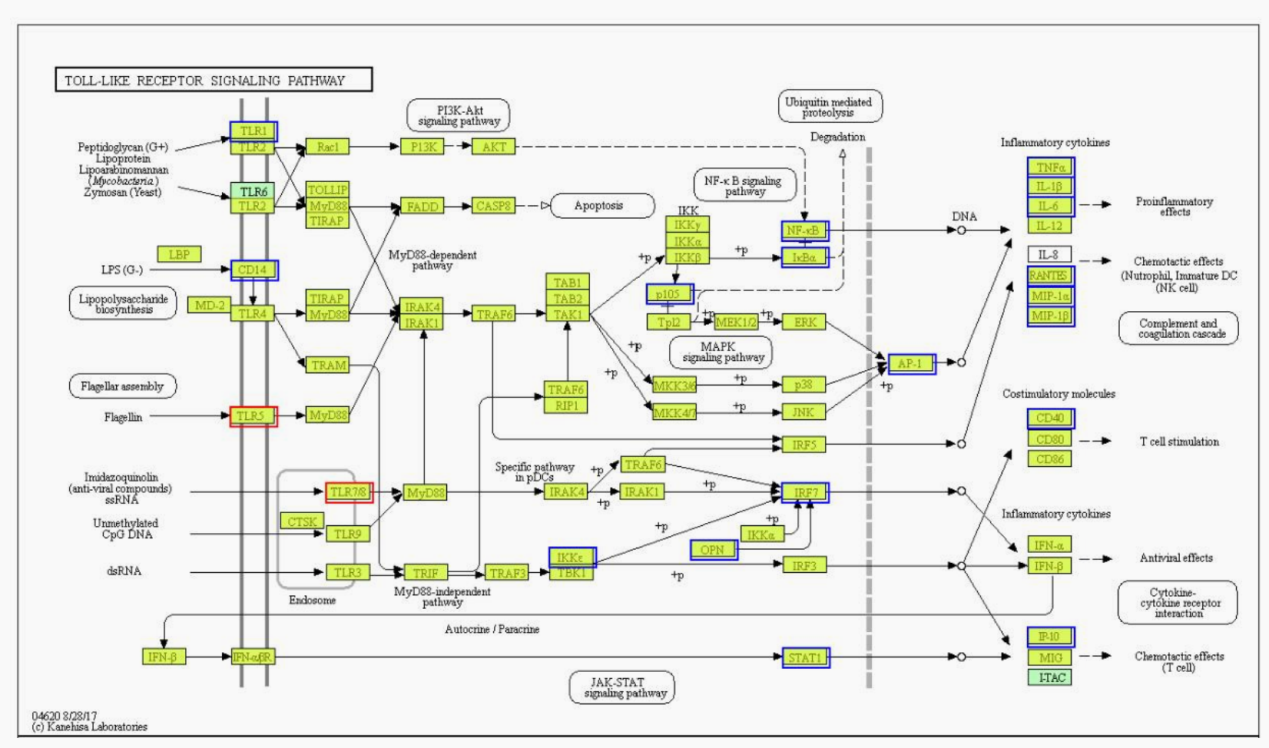


Fig. S2. Effect of LT15 on the toll-like receptor pathway in macrophages
